# Gel-Based Suspension Medium Used in 3D Bioprinting for Constructing Tissue/Organ Analogs

**DOI:** 10.3390/gels10100644

**Published:** 2024-10-10

**Authors:** Yang Luo, Rong Xu, Zeming Hu, Renhao Ni, Tong Zhu, Hua Zhang, Yabin Zhu

**Affiliations:** 1 Health Science Center, Ningbo University, Ningbo 315211, China; 2 Research Institute of Smart Medicine and Biological Engineering, Ningbo University, Ningbo 315211, China

**Keywords:** 3D bioprinting, gel-based suspension medium, tissue/organ analogs

## Abstract

Constructing tissue/organ analogs with natural structures and cell types in vitro offers a valuable strategy for the in situ repair of damaged tissues/organs. Three-dimensional (3D) bioprinting is a flexible method for fabricating these analogs. However, extrusion-based 3D bioprinting faces the challenge of balancing the use of soft bioinks with the need for high-fidelity geometric shapes. To address these challenges, recent advancements have introduced various suspension mediums based on gelatin, agarose, and gellan gum microgels. The emergence of these gel-based suspension mediums has significantly advanced the fabrication of tissue/organ constructs using 3D bioprinting. They effectively stabilize and support soft bioinks, enabling the formation of complex spatial geometries. Moreover, they provide a stable, cell-friendly environment that maximizes cell viability during the printing process. This minireview will summarize the properties, preparation methods, and potential applications of gel-based suspension mediums in constructing tissue/organ analogs, while also addressing current challenges and providing an outlook on the future of 3D bioprinting.

## 1. Introduction

The urgent need to repair damaged human tissues/organs necessitates the design of tissue/organ analogs with physiological structures and functions for in vivo transplantation [1]. Tissue engineering scaffolds, which combine biomaterials, seed cells, and bioactive factors, have been widely used for the repair of damaged tissues/organs [2,3]. However, these tissue engineering scaffolds are unable to mimic the complex and intricate geometrical structures of in vivo tissues/organs, nor can they deposit multiple types of cells in their corresponding spatial locations. These limitations are detrimental to the restoration of the natural structure and function of tissues/organs.

**Figure 1:**
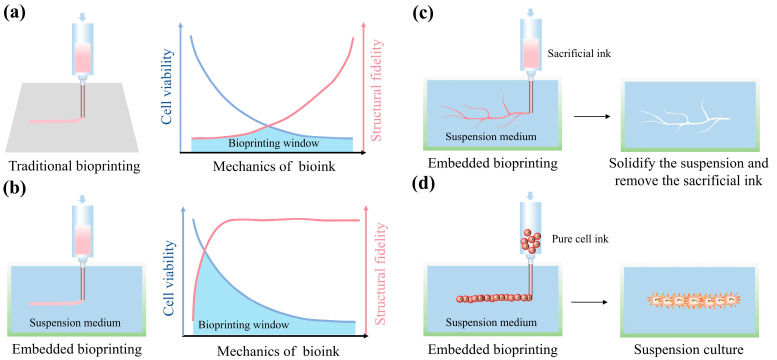
Three-dimensional bioprinting based on gel-based suspension mediums offers a broad printing window and more printing methods compared with traditional printing. The scheme and printing window (considering cell viability and structural fidelity) of traditional bioprinting (**a**) and the embedded bioprinting based on gel-based suspension mediums (**b**). Schematic diagram of bioprinting using a sacrificial ink (**c**) and a pure cell ink (**d**) within a gel-based suspension medium.

Extrusion-based 3D bioprinting has been employed in the engineering of functional tissues/organs because it can accurately replicate the geometrical shapes of natural tissues/organs [4,5]. This technique not only facilitates the creation of transplantable tissues/organs analogs, but also serves in the establishment of in vitro disease models. During the printing process, seed cells are encapsulated within the bioink and are precisely deposited in specific spatial locations as the bioprinter shapes the structures [6]. Traditional extrusion-based 3D bioprinting often requires extrusion filaments with high mechanical properties to shape complex geometrical structures with high fidelity. However, the bioinks with high mechanical properties are not suitable for cell encapsulation, as they often lead to cell death during the printing process or restrict cell growth, proliferation, and self-organization [7,8]. Furthermore, the high shear stress and the prolonged residence time during the printing process will lead to a decrease in cell viability [9,10]. Although replacing bioinks with gel-based materials of lower mechanical strength favors cell survival, the printed geometrical structures often collapse, losing their ability to replicate the natural tissue/organ architecture [11]. Therefore, researchers often introduce additional additives or interpenetrating network hydrogels composed of multiple hydrogels into bioinks to achieve better structural integrity post-printing. For example, Zhang et al. [12] introduced cellulose into the bioink, while Yin et al. [13] used methacrylated gelatin (GelMA) and gelatin as the main components of the bioink. Figure 1a vividly illustrates the schematic diagram of traditional bioprinting and the printing window.

The advent of gel-based suspension mediums has reconciled the conflict between the weak mechanical properties of bioinks and their high-fidelity geometrical structures [14,15,16]. This innovation allows extrusion-based 3D bioprinting to be performed not on a platform, but by embedding the printing nozzle into the gel-based suspension medium. Thus, this kind of printing is also called embedded bioprinting. The extruded filaments from the printing nozzle are received by the suspension medium, which supports the filaments, enabling them to maintain specific spatial positions and ultimately construct high-fidelity geometrical structures [17,18]. This approach enables bioinks with weak mechanical properties to form high-fidelity geometric structures, thereby expanding the bioprinting window (Figure 1b). Additionally, the gel-based suspension medium broadens the range of bioprinting techniques. For instance, when combined with sacrificial inks, it allows for the creation of spatial conduit structures (Figure 1c) [19]. It also supports the printing of pure cell inks, accelerating tissue self-organization (Figure 1d) [20].

The gel-based suspension medium has also exhibited superiority in printing geometries with lower constraints, smaller dimensions, and higher resolutions, which is crucial for shaping fine biomimetic structures [21]. Additionally, performing 3D bioprinting within suspension mediums allows for the use of low-viscosity bioinks, accelerating the biological process of cellular organization, which is highly beneficial for constructing tissue/organ analogs in vitro [22,23]. Therefore, this paper reviews the properties of gel-based suspension mediums, their preparation, and their manufacturing potential in 3D bioprinting. It further discusses the challenges and future prospects of using 3D bioprinting to create mature tissues/organs, aiming to provide valuable references for the construction of tissue/organ analogs in vitro.

## 2. Properties of the Gel-Based Suspension Medium

Gel-based suspension mediums are polymer solutions or sols that support bioinks during the 3D printing process. These mediums are primarily classified into three types, corresponding to the three needs of extrusion-based bioprinting. The first type of suspension medium serves merely as an auxiliary material for 3D bioprinting, used to expand the printing window. This type of suspension medium is typically removed after printing and does not become part of the construct or support subsequent culture. The second type of suspension medium becomes part of the construct. This category of gel-based suspension medium is not removed after bioprinting and forms a stable structure through further photocrosslinking, facilitating transfer along with the construct. The third type of suspension medium serves as a subsequent culture material. This gel-based suspension medium provides nutritional support to seeded cells after bioprinting, mainly applied in the printing of high-density cell inks or pure cell inks. In this chapter, we review the general properties of gel-based suspension mediums and the special properties of the three aforementioned types.

### 2.1. Rheological Properties

#### 2.1.1. Yield Stress

**Figure 2:**
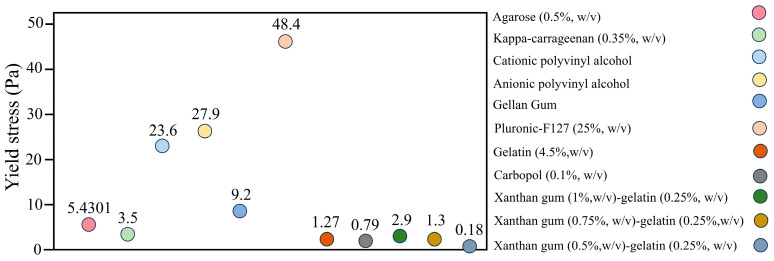
The yield stress of some common gel-based suspension mediums.

The gel-based suspension medium must function as a yield stress fluid, which is characterized by solid-like properties when no stress is applied and a transition to liquid-like behavior when subjected to stress exceeding a critical threshold [24,25]. During the 3D bioprinting process, the critical stress of the gel-based suspension medium should be significantly lower than the stress generated by the nozzle movement. This ensures that the suspension medium around the nozzle undergoes a solid-to-liquid transition, facilitating the extrusion of the bioink and thereby enhancing print quality and precision. Based on the existing literature [26,27,28,29], Figure 2 summarizes the yield stress values of some common gel-based suspension mediums.

#### 2.1.2. Shear Thinning

The gel-based suspension medium must also exhibit shear thinning fluid properties, which are essential for effective embedded 3D bioprinting. During the 3D bioprinting process, the moving nozzle applies a shear stress to the surrounding suspension medium. This reduces the viscosity of the suspension medium, facilitating the extrusion of smooth filaments [30]. Naturally, the faster the nozzle moves, the higher the shear rate, leading to a more significant reduction in the suspension medium’s viscosity. Table 1 summarizes the viscosity values of some common suspension mediums as they vary with shear rate, demonstrating their shear thinning behavior.

#### 2.1.3. Self-Healing

**Figure 3:**
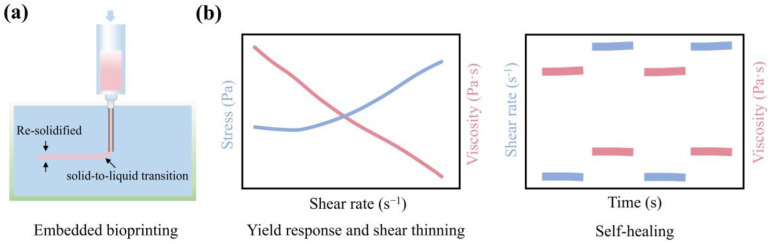
Rheological properties of the gel-based suspension medium. (**a**) The schematic diagram illustrates the state changes during the 3D printing process. The suspension medium near the nozzle transitions from a solid-like to a liquid-like state, facilitating the extrusion of bioinks. Once the nozzle moves away, the liquid-like suspension medium re-solidifies, trapping the bioink in a defined spatial position. (**b**) Representative rheology of a qualified suspension medium, including yield response, shearthinning, and self-healing.

The gel-based suspension medium undergoes a solid-to-liquid transition when subjected to the stress generated by the nozzle movement. When the bioink is extruded into the suspension medium and the applied stress is removed, the suspension medium transitions back from having liquid-like to solid-like properties (Figure 3a) [33]. This self-healing characteristic anchors the extruded bioink in its original spatial position, promoting the creation of high-fidelity geometric structures. Additionally, the self-healing time should be as short as possible to effectively prevent the diffusion of bioinks. Zhang et al. [27] reported a self-healing time of ~5 s for a kappa-carrageenan (0.35%, *w*/*v*) suspension medium, while Scalzone et al. [34] reported that the self-healing time of a Pluronic-F127 (PF-127, 40%, *w*/*v*) suspension medium does not exceed 10 s.

The gel-based suspension medium must possess these specific rheological properties to facilitate the extrusion of bioink without diffusion and provide suspension support, ensuring the smooth movement of the nozzle and preventing clogging. The representative rheological results of a qualified suspension medium are shown in Figure 3b.

### 2.2. Biocompatibility

The suspension medium in direct contact with the bioink can influence the viability of the encapsulated cells, as these two mediums can exchange simple water-soluble substances. Therefore, the gel-based materials and additional additives used for the suspension medium should remain non-toxic. Currently, gel-based suspension mediums used for 3D bioprinting are primarily composed of biocompatible hydrogels, such as gelatin [35], gellan gum (GG) [36], xanthan gum (XG) [29], and PF-127 [37].

### 2.3. Transparency

A transparent suspension medium allows us to monitor the printing process, making the deposition of bioink and the integrity of layer-by-layer printing visible. Moreover, to solidify the printed structures, soft bioinks often require further ionic complexation or UV crosslinking. For bioinks that need UV crosslinking, a transparent suspension medium is particularly important, as it does not obstruct the penetration of UV light.

### 2.4. Specifical Properties

#### 2.4.1. Removable

Most applications require the printed structures to be released from the suspension medium; therefore, the suspension medium should be removable after bioprinting. Research [38] has shown that the suspension medium remains more or less on the printed structures, and the removal efficiency primarily depends on the dissolution properties of the suspension medium. Additionally, gel-based suspension mediums obtained through chemical crosslinking leave residues tens of times higher than those obtained through physical crosslinking. These residual suspension mediums adhere to the surface of the constructs and can even penetrate into the interiors. Over the course of long-term culturing, these suspension mediums are gradually removed, leading to surface roughening and the formation of cavities within the constructs. These changes can reduce the mechanical strength of the constructs.

#### 2.4.2. Photopolymerizable

Certain specialized applications, such as the construction of spatial conduit structures, require the gel-based suspension medium to ultimately become part of the construct. To meet this need, the suspension medium must undergo further photocrosslinking to form a stable structure. Therefore, photopolymerizable gel precursor solutions often become a crucial component of the suspension medium. To convert photopolymerizable gel precursor solutions into photopolymerizable suspension mediums, researchers often promote the precursor solutions to acquire the desired rheological properties by regulating temperature and introducing additives [39,40].

#### 2.4.3. Supportive Suspension Culture

Some studies require high-density cell-laden bioinks or pure cell inks for the construction of tissues/organs. For these applications, the suspension medium must remain after printing to provide structural support for the printed constructs. Moreover, the suspension medium must supply sufficient nutrients to maintain cell viability and promote tissue development [41]. For example, Brassard et al. [20] printed organoid-forming stem cells into an extracellular matrix-like suspension medium. After cultivation, they obtained centimeter-scale tissues featuring self-organizing characteristics, including lumens, branching vasculature, and tubulated intestinal epithelium. It is noteworthy that the suspension medium used for cell culturing primarily consists of particulate microgels. Nutrients are stored within the interstices between the microgel particles and freely diffuse throughout the culture system. Cells uptake nutrients and discharge metabolic waste through these interstitial spaces to maintain viability [24]. Moreover, the stress exerted by the suspension medium on the cell surface (yield stress) is less than the cytoskeletal force during cell division and the actin polymerization force during migration, thereby not impeding the proliferative and migratory capabilities of the cells. On the contrary, the presence of this yield stress limits stress accumulation during proliferation and migration, potentially aiding in the prolonged vitality of cells within a 3D environment [24].

## 3. Preparation of Suspension Mediums in the Form of Microgels

Gel-based suspension mediums primarily exist in two forms. One is the critical state between the liquid and gel phases. This type of suspension medium is prepared by controlling the temperature or introducing additives, making the method straightforward [39,42]. The other form is particulate microgels [43,44]. This type of gel-based suspension medium is currently the most widely used in 3D bioprinting, and its preparation involves the production of a large number of particulate microgels. Here, we mainly discuss the preparation process of microgel-based suspension mediums.

### 3.1. Mechanical Blending

Mechanical blending is the simplest and most commonly used method for obtaining microgel suspension mediums. A single block of gelatin hydrogel, after processes such as blending and centrifugation, transforms into microgels with a mean feret diameter of 55.3 ± 2 μm. When these microgels are resuspended, a microgel suspension medium is obtained, capable of achieving a printing resolution of 199 ± 41 μm [45]. Additionally, mechanical blending has been employed in the preparation of polyvinyl alcohol (PVA) suspension medium [28], XG suspension medium [46], agarose suspension medium [26], and GG suspension medium [47].

Microgels prepared in this manner generally have large and unevenly distributed particle sizes, often resulting in low bioprinting resolutions [48]. To improve bioprinting resolution, researchers have modified the mechanical blending method for preparing microgels. For example, Xie et al. [49] added trisodium citrate during the preparation of gellan gum suspension medium, inhibiting microgel aggregation and resulting in uniformly distributed gel particles approximately 30 μm in size. Zhang et al. [27,30] prepared submicron-sized microgels by pulverizing low-crosslinked kappa-carrageenan.

### 3.2. Coacervation

The coacervation method involves obtaining microgel particles by continuously stirring a gel liquid in a coagulated state. This method was proposed by Lee et al. [50] as an improvement over the mechanical blending method for preparing gelatin-based suspension mediums. Specifically, gelatin is dissolved in a composite solvent, and by gradually cooling while stirring, the gelatin molecules are induced to coagulate into uniformly shaped gelatin microspheres with a particle size of ~25 μm. This microgel suspension medium can reliably print filaments ranging from 200 μm to 20 μm in width. Similarly, Noor et al. [51] dissolved sodium alginate (ALG) in a calcium carbonate suspension, and by continuously stirring while slowly adjusting the pH of the suspension, the gradual release of Ca^2+^ was induced to complex with the ALG, resulting in a microgel. Additionally, agarose suspension mediums can also be obtained using this method [52].

### 3.3. Flash-Solidification

The flash-solidification method differs slightly from the coacervation method. It involves dispersing the precursor gel solution into small droplets and then rapidly gelling them to obtain a large number of microgels. Sreepadmanabh et al. [53] prepared agarose microgels with a particle size of 100 ± 50 µm by injecting hot agarose solution into a cold continuous phase solvent under rapid stirring. The hot agarose solution was first broken into fine droplets and then solidified into microgel particles under low temperature conditions.

### 3.4. Air-Assisted Co-Axial Jetting

This technique utilizes air pressure to eject the gel precursor solution from a co-axial needle into fine droplets, which are then converted into microgels using photocrosslinking or ionic complexation methods. Pal et al. [54] employed this method to prepare ALG microgels for use as suspension mediums, with the microgel particle size primarily determined by air pressure. When the pressure increased from 40 kPa to 180 kPa, the microgel particle size decreased from 202 ± 14 μm to 49 ± 4 μm.

**Figure 4:**
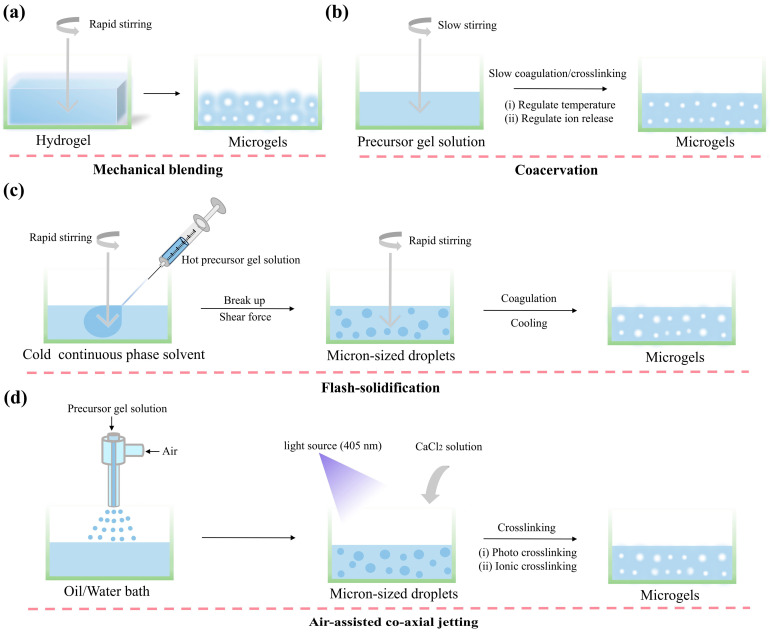
Preparation of gel-based suspension medium. (**a**) Mechanical blending; (**b**) coacervation; (**c**) flash solidification; and (**d**) air-assisted co-axial jetting.

The particle size, shape, and dispersibility of the microgels comprising the gel-based suspension medium are closely related to the fidelity and resolution of bioprinting [55,56]. Typically, the larger particle sizes, irregular shapes, and poor dispersibility of microgel mediums often result in rough and uneven printed filaments, as the bioink easily infiltrates the gaps between the microgels [31]. Therefore, the goal in developing new suspension medium is to produce microgels with a uniform particle size, regular shape, and good dispersibility. The above sections summarized four methods for preparing gel-based suspension mediums (Figure 4), and their advantages and limitations are evaluated below (Table 2).

## 4. Gel-Based Suspension Mediums Used in Constructing Tissue/Organ Analogs

Three-dimensional bioprinting provides a technology for the construction of biomimetic tissues/organs, thereby opening up new avenues for repairing or replacing diseased tissues/organs. The advent of gel-based suspension mediums has expanded the window for bioprinting, allowing for the application of low-viscosity, highly flowable bioinks. These bioinks are deposited within the suspension medium to form high-resolution and high-fidelity biomimetic structures. Here, we summarize the cases reported in the literature, where a gel-based suspension medium was used to construct tissue/organ analogs, and discuss the significance of these works.

### 4.1. Cornea

The cornea is a transparent membrane located at the front of the eye, responsible for allowing light to enter and for protecting the eyeball. However, corneal diseases or injuries are among the leading causes of blindness in patients [57]. Although this problem can be addressed through corneal transplantation, the shortage of donor corneas prevents most patients from regaining their sight [58]. Using 3D bioprinting technology to create corneal substitutes has become a research hotspot in corneal tissue engineering. The cornea is a transparent curved membrane, with surface roughness at the submicron level [59]. Researchers find it challenging to directly print curved structures on a flat platform, often requiring the assistance of hemispherical molds that resemble the natural curvature of the cornea [60]. Nonetheless, adjusting the composition of bioinks or the environmental temperature is necessary to achieve a smooth surface on the curved membrane [60,61]. By utilizing suspension mediums, researchers can accurately replicate the microscopic scale of the cornea. Zhang et al. [62] used GelMA as a bioink to print corneal structures in a suspension medium composed of nanoclay and PF-127. This work effectively eliminated the rough traces on the surface of the engineered cornea by controlling the interlayer spacing and the overlap ratio of the bioink, rendering the engineered cornea smooth and transparent.

### 4.2. Vasculature

The construction of vascular networks is a prerequisite for the survival of tissue/organs, as they are responsible for the exchange of gasses and nutrients [63]. As hollow structures, there are various methods to print biomimetic vascular networks, including co-axial bioprinting [64], 3D printing based on sacrificial materials [65], and inkjet printing [66]. Using suspension mediums, hollow structures can be easily obtained [67]. For example, researchers utilized artificial intelligence to segment and 3D print coronary arteries from medical images, successfully fabricating branched coronary artery structures. The significance of this research lies in its potential to create patient-specific vascular grafts [68]. Kreimendahl et al. [69] introduced human umbilical vein endothelial cells and human dermal fibroblasts into constructs composed of fibrinogen and hyaluronic acid (HA), discovering vascularization within the constructs. More interestingly, Machour and Szklanny et al. [70,71] developed engineered vascular flaps suitable for in vivo transplantation. The research team first used recombinant human collagen methacrylate (rhCollMA) as a bioink to print microvascular networks in a gelatin-based suspension medium, then integrated the microvascular networks with vascular scaffolds, resulting in engineered vascular flaps that achieved blood flow reconstruction in vivo.

### 4.3. Menisci

The menisci are responsible for shock absorption in the knee joint and are highly susceptible to injury. However, due to the lack of vascularization in their inner regions, they do not heal well [72]. Prendergast et al. [73] developed a tissue patch for meniscal tears by incorporating norbornene-functionalized hyaluronic acid (NorHA) microfibers into a bioink (GelMA), which guided the oriented growth of meniscal fibrochondrocytes and promoted the maturation of meniscus-like anisotropic structures. Although the use of 3D bioprinting technology to construct biomimetic menisci in vitro offers a promising approach for meniscal repair [74,75], it remains challenging to shape a meniscus that possesses both highly vascularized fibrous connective tissue (outer region) and avascular cartilage tissue (inner region). Terpstra et al. [76] printed a biomimetic meniscus in a GG-based suspension medium, precisely controlling the anisotropic distribution of vascular networks within the meniscus. This engineered meniscus successfully mimicked the highly vascularized outer region and the avascular inner region of the natural meniscus.

### 4.4. Neuron Networks

Three-dimensional neural cultures provide an excellent model for studying the signaling mechanisms of neuron growth, differentiation, and neural network formation [77]. With the aid of 3D bioprinting technology, these models can be readily obtained [78,79]. The advent of gel-based suspension mediums has created more opportunities for the in vitro construction of neural networks. Hirano et al. [39] seeded sensory neurons, induced from human pluripotent stem cells, into 3D network channels and successfully cultivated sensory neuron networks. These 3D network channels were constructed by removing sacrificial ink (gelatin) from a GelMA suspension medium. The resulting neural networks not only serve as models for studying neural signaling mechanisms, but also hold potential for integration into engineered tissue/organ equivalents. These sensory-capable neural implants could potentially connect with host nerves in injured areas to regenerate sensory functions. Moreover, Wang et al. [80] integrated neural networks into vascular structures, constructing neurovascular units in vitro. This in vitro model is valuable for neuropharmaceutical screening and the establishment of specific brain disease models.

### 4.5. Skeletal Muscle

Skeletal muscle is composed of aligned muscle fibers. Researchers have developed some 3D fiber bundle structures in vitro for skeletal muscle tissue regeneration [81,82]. However, they overlooked the fact that blood vessels and motor nerves are interspersed among the muscle fibers in skeletal muscle. Therefore, constructing skeletal muscle mimics by merely densely stacking highly aligned muscle fibers is insufficient; it is necessary to integrate both blood vessels and nerves [83,84]. Hassan et al. [85] integrated a multi-channel microfluidic device with embedded 3D printing technology to print skeletal muscle fiber mimics in a gelatin-based suspension medium. These mimics were composed of multiple fibers with a core–shell structure, where muscle cells formed the core and vascular endothelial cells constituted the shell. This combined structure perfectly replicated the segregated and aligned fibers of natural skeletal muscle, with the inclusion of blood vessels. However, there is a scarcity of literature on the in vitro construction of biomimetic skeletal muscle that includes both neural networks and vascularization.

### 4.6. Heart

Printing a transplantable heart in vitro poses challenges for researchers at this current stage. Presently, the establishment of in vitro heart models is primarily aimed at surgical simulation and training [86]. Mirdamadi et al. [87] constructed a full-sized heart model that not only possesses high fidelity but also has sufficient toughness for suturing and blood perfusion. Although this work does not involve cells, it holds significant importance for the development of surgical models and the training of surgeons. Additionally, the construction of heart models in vitro that exhibit contraction and electrical signaling is considered a substitute for animal models and can serve as a platform for drug testing [88]. Esser et al. [89] incorporated human-induced pluripotent stem cell-derived cardiomyocytes into collagen/hyaluronic acid bioinks, printing ring-shaped and ventricular-shaped cardiac tissues that can spontaneously contract and respond to external drug stimulation.

### 4.7. Brain

Similar to heart models, the in vitro construction of full-sized human brain models aids clinicians in conducting standardized training before brain surgery. Hua et al. [40] printed a full-sized human brain model using a photopolymerizable suspension medium. They selected gelatin and alginate (ALG) as sacrificial inks to print the brain contours. After the internal brain model was crosslinked by light, the external contours were removed, resulting in a complete brain model.

These gel-based suspension medium-printed tissue/organ analogs, and the significance of these works, are summarized in Table 3.

## 5. Challenge

While gel-based suspension mediums facilitate embedded bioprinting, they still present challenges that researchers need to overcome. Currently, most suspension mediums, whether purchased or synthesized by researchers, are discarded after printing, wasting time and material costs [90]. Therefore, developing recyclable and reusable gel-based suspension mediums is crucial for minimizing waste in the 3D bioprinting process. Additionally, the suspension medium cannot be completely removed after printing. Although these suspension mediums are made from highly biocompatible materials and do not exhibit cytotoxicity, it is unknown whether these residual suspension mediums obstruct the exchange of substances between cells and their external environment or block the fine pore/channel structures within the constructs. These factors could potentially limit cell adhesion, proliferation, differentiation, and self-organization within the constructs.

For the creation of tissue/organ equivalents through bioprinting, we still face the challenge of integrating vasculature and nerves [84,91,92,93]. Despite researchers’ significant efforts to replicate the structures of natural tissues/organs, we are still far from creating equivalents that possess autonomous blood circulation and responses to physiological stimulation. Furthermore, using 3D printing to cultivate physiologically relevant human-scale models in vitro requires additional efforts. Constructing large organs necessitates not only the integration of vascular and neural networks but also the involvement of various cell types. These cells are anisotropic and are distributed within the tissues/organs, exchanging stimulatory signals without interfering with each other’s execution. These requirements guide us in preparing multi-channel cell-laden bioinks and precisely controlling spatial deposition during the printing process.

## 6. Conclusions and Perspectives

Three-dimensional bioprinting, which can replicate the intricate structures of natural tissues/organs, has become an essential method for constructing biomimetic tissues/organs. The emergence of gel-based suspension mediums has propelled the further development of bioprinting technology by resolving the conflict between soft bioinks and high-fidelity biomimetic structures, promoting the precise deposition of cells within constructs. Soft geometric scaffolds enable rapid cell proliferation and differentiation, thereby facilitating the development and maturation of biomimetic tissues/organs. This review summarizes the unique properties of gel-based suspension mediums, their preparation methods, and their potential in constructing biomimetic structures, providing researchers with a deeper understanding.

In the future, 3D bioprinting technology should be integrated with machine learning and artificial intelligence modeling to create patient-specific tissue/organ analogs, rather than merely segmenting and printing existing tissue/organ models. Artificial intelligence can assist in the segmentation of complex medical images, while machine learning enables real-time defect detection in 3D printing, thereby ensuring the geometrical accuracy of the constructs [94,95]. Furthermore, 3D bioprinting should incorporate a temporal dimension, gradually evolving into 4D bioprinting. For instance, researchers could continue to culture tissue/organ constructs in suspension mediums post-printing, promoting their development into specific disease models or more physiologically accurate tissue/organ analogs by regulating the release of drugs, hormones, and growth factors from the suspension medium at specific time points. For bioinks, 4D bioprinting necessitates the use of smart materials that are responsive to external stimuli such as light, heat, electricity, magnetism, PH, etc. Researchers can manipulate changes in the external environment to regulate the properties of the constructs, including morphology, surface activity, solubility, and configuration [96]. These modifications induce specific physiological and biochemical changes in the encapsulated cells, including targeted differentiation, matrix production, and tissue maturation. Additionally, biosensors can be employed to evaluate the physiological events of the tissue/organ analogs, which is invaluable for the construction of disease models, drug screening, and therapy in vitro.

The advancement of gel-based suspension mediums has propelled the development of biomimetic tissue/organ construction through 3D bioprinting, marking just the beginning. We believe that one day, this technology will be truly utilized in clinical applications.

## Figures and Tables

**Table 1:** The shear thinning behavior of some common gel-based suspension mediums.

Gel-Based Suspension Medium	Shear Rate (1/s)	Viscosity (Pa·s)	Ref.
Kappa-carrageenan (0.35%, *w*/*v*)	From 10^−3^ to 10^1^	~From 10^4^ to 10^1^	[27]
Pluronic-F127 (25%, *w*/*v*)	From 10^−3^ to 10^3^	~From 10^4^ to 10^0^	[29]
Gelatin (4.5%, *w*/*v*)	From 10^−3^ to 10^3^	~From 10^3^ to 10^−1^	
Carbopol (0.1%, *w*/*v*)	From 10^−3^ to 10^3^	~From 10^3^ to 10^−1^	
Alginate	From 10^−1^ to 10^0^	~From 250 to 50	[31]
Agarose (0. 25 wt%)	From 10^−2^ to 10^2^	~From 10^1^ to 10^−1^	[32]

**Table 2:** Evaluation of preparing methods using gel-based suspension mediums.

Methods	Advantages	Limitations
Mechanical blending	Simple operation and short preparation time	Microgels were prepared with random sizes and irregular shapes. The introduction of additives or the selection of gels with low crosslinking degrees were required.
Coacervation	The microgels exhibit regular shapes, small particle sizes, and uniform distributions.	Long preparation time
Flash-solidification	Short preparation time	The microgels have large and uneven particle sizes.
Air-assisted co-axial jetting	Short preparation time	The size of the microgels is related to the air pressure; achieving small gel particles is challenging.

**Table 3:** Gel-based suspension medium used in constructing tissue/organ analogs.

Biomimetic Structures	Gel-Based Suspension Medium	Bioink	Significance	Ref.
Cornea	Nanoclay/PF-127	GelMA	Eliminating surface roughness caused by layered morphology	[62]
Vasculature	PF-127	ALG/glucomannan	Customing vascularized grafts	[68]
	gelatin	fibrinogen-HA	Constructing vascular networks in vitro	[69]
	gelatin	rhCollMA	Constructing transplantable vascular flaps	[70,71]
Menisci	agarose	GelMA/NorHA microfibers	Repairing meniscus tear	[73]
	GG	Fibrinogen/gelatin	Spatial control of the vascular network in menisci	[76]
Neuron networks	GelMA	Gelatin	Integrated into engineered tissue/organ equivalents	[39]
	Gelatin	ALG/Collagen, Collagen	Disease modeling/drug screening	[80]
Skeletal muscle	Gelatin	GelMA/ALG	Developing a combined approach of multi-material and embedded bioprinting	[85]
Heart	Gelatin	ALG	Surgical modeling and training	[87]
	Gelatin/gum arabic	Collagen/HA	Drug screening/ organ repair	[89]
Brain	PEGDA/Nanoclay/PF-127	Gelatin/ALG	Surgical modeling and training	[40]

## Data Availability

Not applicable.
